# Hydrolysis of doped conducting polymers

**DOI:** 10.1038/s42004-020-00404-y

**Published:** 2020-11-04

**Authors:** Vithyasaahar Sethumadhavan, Kamil Zuber, Christopher Bassell, Peter R. Teasdale, Drew Evans

**Affiliations:** 1 Future Industries Institute, University of South Australia, Mawson Lakes, SA 5095 Australia; 2 UniSA STEM, University of South Australia, Mawson Lakes, SA 5095 Australia

**Keywords:** Polymers, Conjugated polymers

## Abstract

Conducting polymers display a range of interesting properties, from electrical conduction to tunable optical absorption and mechanical flexibility, to name but a few. Their properties arise from positive charges (carbocations) on their conjugated backbone that are stabilised by counterions doped in the polymer matrix. In this research we report hydrolysis of these carbocations when poly(3,4-ethylenedioxy thiophene) is exposed to 1 mM aqueous salt solutions. Remarkably, two classes of anion interactions are revealed; anions that oxidise PEDOT via a doping process, and those that facilitate the S_N_1 hydrolysis of the carbocation to create hydroxylated PEDOT. A pKa of 6.4 for the conjugate acid of the anion approximately marks the transition between chemical oxidation and hydrolysis. PEDOT can be cycled between hydrolysis and oxidation by alternating exposure to different salt solutions. This has ramifications for using doped conducting polymers in aqueous environments (such as sensing, energy storage and biomedical devices).

## Abstract (web summary)

The properties of conducting polymers are known to be controlled by counterions, but this remains poorly understood. Here the aqueous hydrolysis and oxidation of PEDOT is studied as a function of counterion pKa.

## Introduction

Materials with tunable electrical, optical and/or thermal properties offer new paradigms in the fabrication of devices. Furthermore, when these material properties respond to changes in the local environment opportunities arise for sensing, detection and/or monitoring applications. One class of material with tunable properties are conducting polymers^1–4^ – polymers consisting of a conjugated (π-bonded) backbone along which positive charges reside that are stabilised by doping counterions. These positive charges predominantly exist as carbocations that are further stabilised by resonance along the π-bonded network. Depending on the level of doping and related oxidation state of the polymer it will have a quinoid or benzenoid character leading to different optical, electrochemical and electrical properties^5,6^. The conducting polymer poly(3,4-ethylenedioxy thiophene) (PEDOT) is considered one of the prototypical members of this class of material, displaying excellent electrical, optical, thermal and mechanical properties^7–11^. This polymer has demonstrated utility in a wide range of applications including neurological sensing^12,13^, thermoelectric harvesters^14,15^, electrochromic displays^16,17^, agricultural sensing^18^, and transparent electrodes^19–21^.

**Fig. 1:**
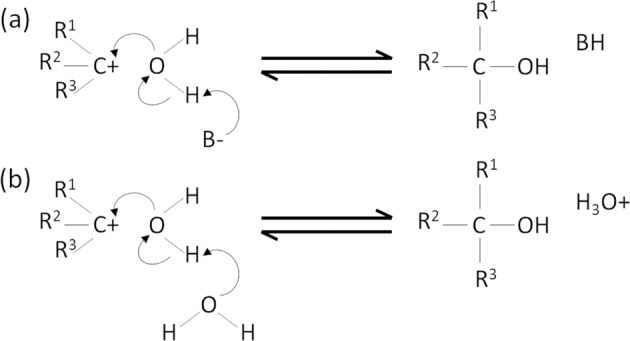
The pathways to hydrolysis of a carbocation. **a** base-catalysed hydrolysis with a base (B^−^) as the catalyst in a concerted mechanism; and **b** water-catalysed hydrolysis by water molecules neighbouring to the water molecule acting as the nucleophile. R^1^, R^2^ and R^3^ represent atoms to which the C is bonded and may or may not be the same atoms depending on the specific molecule in question.

In organic chemistry, the carbocation has been widely studied as an intermediate in a variety of addition, substitution and elimination reactions^22^. Much of the research into the hydrolysis/solvolysis of carbocations focuses on the rate limiting step of initially forming and stabilising the carbocation^22^, whether the carbocation forms as a consequence of protonation of an alkene (−C=C– → –(CH)–C^+^–), or from dissociation via separation of a good leaving group such as a halide (−C–Br → −C^+^ + Br^−^). Once the carbocation is formed, it can be utilised in a wide range of reactions^22^. In the specific case of hydrolysis of the carbocation, water acts as a nucleophile to bond with the carbocation. The pathway for this depends on the molecule catalysing the reaction. For the base-catalysed pathway in Fig. 1a, the base deprotonates water at the same time the oxygen lone pair bonds with the carbocation^23^. In the work of Ritchie et al. they provided a strong argument to support the case that general base-catalysed hydrolysis proceeds via a concerted step; as shown in Fig. 1b; the water bonds to the carbocation and is deprotonated in one step (therefore the intermediate hydronium ion is not observed)^24^. They state that “…g*eneral base catalysis is characteristic of the reactions of water with any cation of measurable stability in aqueous solution*.” Alternatively, water itself may act as the catalyst (so called water-catalysed in Fig. 1b) to deprotonate the nucleophile water molecule, generating a bound hydroxyl group and a free hydronium ion.

**Fig. 2:**
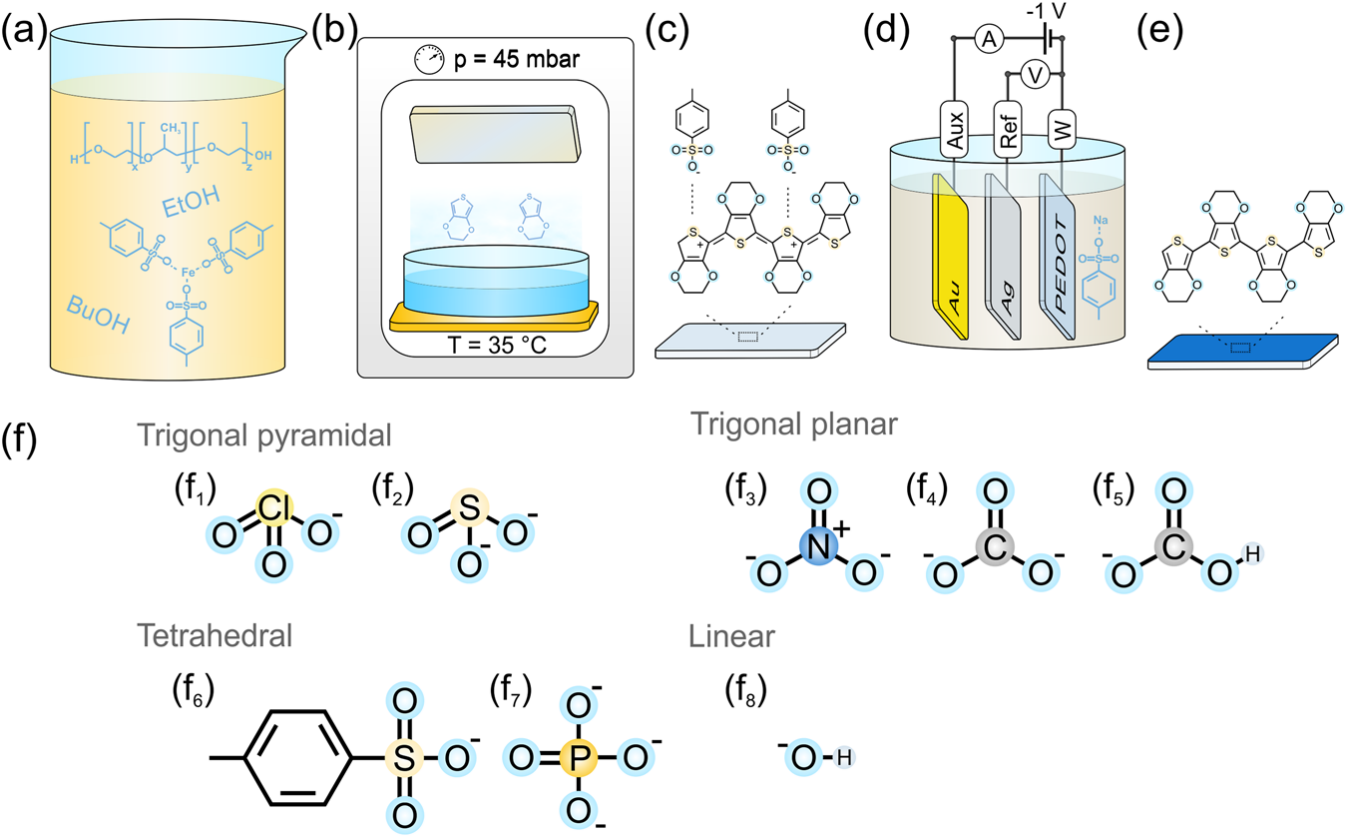
Fabrication of electrochemically reduced PEDOT:Tos samples and their anion exposure. Synthesis of PEDOT:Tos via vapor phase polymerisation process: **a** Oxidant solution is spin coated onto a substrate and **b** exposed to the monomer (EDOT) vapour in vacuum environment. The PEDOT:Tos film (**c**) is then rinsed with ethanol and **d** electrochemically reduced (ER) in a three electrode cell using 1 mM NaTos. ER PEDOT:Tos (**e**) is then exposed to solutions of single secondary dopants (**f**) of various geometries: trigonal pyramidal (f_1_) chlorate (ClO_3_^−^) and (f_2_) sulfite (SO_3_^2−^); trigonal planar (f_3_) nitrate (NO_3_^−^), (f_4_) carbonate (CO_3_^2−^) and (f_5_) bicarbonate (HCO_3_^−^); tetrahedral (f_6_) p-toluenesulfonate (Tos) and (f_7_) phosphate (PO_4_^3−^); and linear (f_8_) hydroxide (OH^−^).

Doped conducting polymers are well described in terms of the type of charge carriers within, i.e. polarons and bipolarons^25^. When these positively charged species reside on carbon atoms in the conjugated backbone, they represent carbocations. In the study here, the polarons and bipolarons are generalised as carbocations thus providing a bridge in the terminology used to describe chemical reactions. In the context of the study herein, PEDOT doped with tosylate (PEDOT:Tos) represents a material of notable carbocation stability, having long lived stable carbocations in high concentration. Put more generally, the class of doped conducting polymers represent a source of carbocation structures where the rate limiting step of carbocation formation has been overcome during the synthesis and doping process. To observe how the stable carbocations interact with water, PEDOT:Tos prepared by vapour phase polymerisation (VPP) was electrochemically reduced (ER) and then exposed to 1 mM aqueous solutions of sodium nitrate (NO_3_^−^), sodium chlorate (ClO_3_^−^), sodium bicarbonate (HCO_3_^−^), sodium sulfite (SO_3_^2−^), sodium carbonate (CO_3_^2−^), sodium phosphate (PO_4_^3−^), and sodium hydroxide (OH^−^). These anions were initially chosen for their diverse properties of geometry, size, charge, and solubility (Fig. 2f). The significance of their base properties was noted subsequently based on observations of initial studies and consequently became the focus for this study. As will be shown in the discussion below, the difference in geometry, size, charge and solubility appear to play insignificant roles compared to their base properties. The experimental results highlight that under certain conditions the prototypical conducting polymer PEDOT:Tos undergoes reaction with the surrounding water. This reaction is facilitated (or not) by the anions present in solution.

## Results and discussion

It is relevant to reiterate that understanding the interactions of conducting polymers with water will underpin their utility in aqueous environments. Prior studies on conducting polymers instruct that different anions in solution will either chemically oxidise or reduce the PEDOT:Tos. Khan et al. reported that PEDOT:Tos exposed to OH^−^ leads to the ion exchange of Tos^−^ with OH^−^ and a commensurate decrease in electrical performance, in polymer chain ordering and chain oxidation level^26^. While the net result of decreasing electrical conductivity is the same, the aforementioned Tos to OH ion exchange is a different mechanism to when polymeric counterions dope the PEDOT (such as polystyrene sulfonate, PSS^−^). In this case exposure to a base such as NaOH leads to insertion of Na^+^ to neutralise the strong acid of PSS^−^ and form Na:^+^PSS^−^ yielding a loss of the carbocation on the PEDOT^27^. In both cases however there is no charge transfer (the PEDOT behaves as a pure capacitor with no Faradaic reactions^28^) and the neutral conjugated PEDOT is formed (that is comparatively lower in electrical conductivity and stronger absorbing in the visible spectra). Almost all these prior studies focus on concentrated salt solutions where the pH is deliberately well away from neutral (i.e. » 10 mM base or acid)^26,29^. In the study herein we employed lower concentrations of salt (1 mM) to study the interaction with PEDOT:Tos at concentrations relevant to applications such as environmental monitoring (i.e. [NO_3_^−^] and [ClO_3_^−^] ≤ 1 mM in ground water^18,30^ and potable water supplies^31,32^), food (i.e. [SO_3_^2−^] « 1 mM^33^), and biological sensing (i.e. [Cl-] « 1 mM in human tissue^34^).

### Response to different anions

**Fig. 3:**
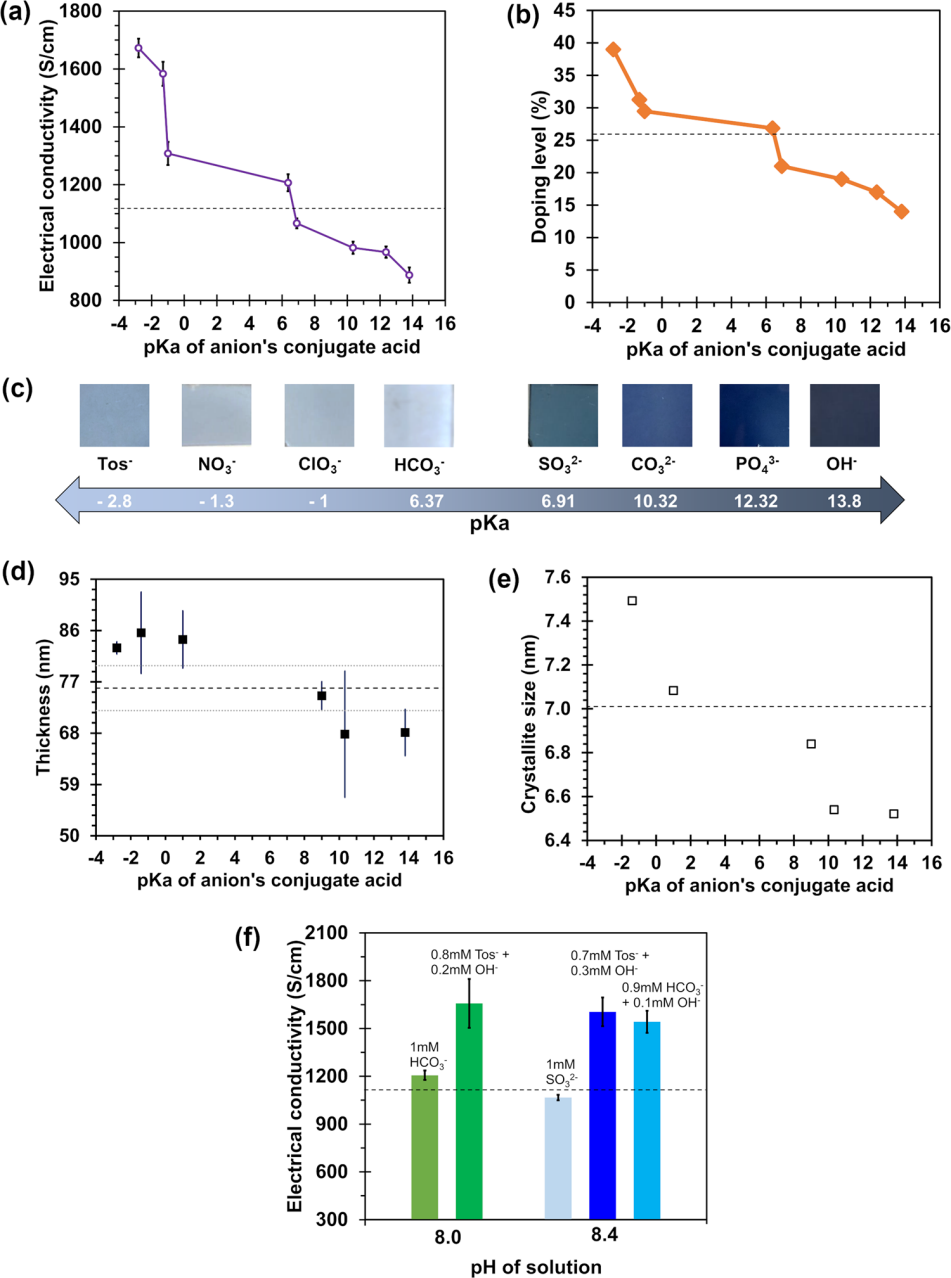
Properties of ER PEDOT:Tos after exposure to salt solutions. **a** Electrical conductivity and **b** doping level of PEDOT:Tos-X as a function of the pKa for the anion’s conjugate acid (dashed line is ER PEDOT:Tos). The data points in **a**, **b** refer to anions listed in **c**. **c** Optical images of PEDOT:Tos-X showing the change in visible absorption (with the conjugate’s pKa) typically associated with changes in the oxidation level of PEDOT. **d** The thickness of the PEDOT:Tos-X samples shown in **c** and used to calculate the electrical conductivity in **a** (dashed line is ER PEDOT:Tos, with ±one standard deviation shown with grey dotted lines). **e** The crystallite domain size determined from XRD spectra for the PEDOT:Tos-X samples (dashed line is ER PEDOT:Tos). **f** Comparison of the electrical conductivity with mixed anion solutions at a constant molarity and fixed pH. Error bars represent ± one standard deviation of measurement across multiple samples.

VPP was used to fabricate thin films of PEDOT:Tos^35,36^, which were then subsequently electrochemically reduced, prior to exposure to the aqueous salt solutions (Fig. 3). The ER PEDOT:Tos acts as a baseline material to observe either the chemical oxidation or reduction (or hydrolysis) of the polymer (though it still contained residual Tos^−^ as shown later in x-ray photoelectron spectroscopy, XPS, spectra). Each of the anions studied herein are defined in terms of the dissociation constant (pKa) of their conjugate acid. The dissociation constants of the conjugate acid for the anions studied is ordered as: Tos^−^ (−2.8)^37^ > NO_3_^−^ (−1.3) > ClO_3_^−^ (−1.0) > HCO_3_^−^ (6.3) > SO_3_^2−^ (6.9) > CO_3_^2−^ (10.4) > PO_4_^3−^ (12.32) > OH^−^ (14.3)^38^.

Figure 3a provides an analysis of the electrical conductivity after exposure to the salt solutions as a function of the pKa of the anion’s conjugate acid (herein referred to as the conjugate’s pKa). When the conjugate’s pKa is low the resultant PEDOT:Tos-X (where X = the anion the polymer was exposed to) has an elevated conductivity. The converse is also observed, when the conjugate’s pKa is high the electrical conductivity decreases. Chemical analysis via XPS (Fig. 3b) reveals that the doping level of counterions (Tos^−^ plus X) within the PEDOT matrix is primarily responsible for the electrical conductivity change—higher doping levels correlate with higher conductivity, and vice versa (*R*^2^ = 0.94, Supplementary Fig. 1). Correlating with these analyses of the PEDOT:Tos-X samples is the optical absorption in the visible region transitioning from near transparent to strongly absorbing as the conjugate’s pKa increases (Fig. 3c). These observations are in good agreement with previous studies exploring the influence of pH on PEDOT:Tos^26^. Indeed, the pH is markedly different for the 1mM binary salt solutions tested. The thickness of the PEDOT:Tos-X samples is presented in Fig. 3d, highlighting that the darker coloured samples are not due to increased thickness. In fact, there is a subtle decrease in thickness for samples exposed to anions with conjugate acid’s having a pKa > 6.4. Figure 3e shows that relative to ER PEDOT:Tos, the crystalline domain size calculated from X-ray diffractograms (Supplementary Fig. 2) increases (decreases) when the conjugate’s pKa is below (above) 6.4. Simulations made recently by Modarresi et al. indicated that PEDOT’s crystallite size increases with increasing oxidation level^39^. This implies that increasing (decreasing) doping level would yield increasing (decreasing) crystallite size, in agreement with the structural changes observed herein. In the studies by Khan et al. the size of the crystalline domains in PEDOT:Tos (determined from XRD) was shown to decrease with both acidic and basic pH relative to pristine conducting polymer^26^. The discrepancy of increasing or decreasing crystallite size at lower pH is hypothesised to arise from the fact that Khan et al. studied much higher salt concentrations (≥10 mM) than the present study (1 mM). To remove the possible pH influence, several solutions were prepared at a constant pH and constant total molarity using mixtures of anions (Fig. 3f). Interesting to note is that the resultant conductivity of the exposed PEDOT:Tos-X varies depending on the specific anions in solution and is somewhat independent of the pH. For example, PEDOT:Tos-Tos/OH is 50% more conductive than PEDOT:Tos-HCO_3_/OH. This suggests that the specific nature of the anion is critical, above and beyond the pH alone. We hypothesise that a competition exists between the base catalysed hydrolysis (loss of carbocations) of the ER PEDOT:Tos and its doping with anions whose conjugate’s have a low pKa.

### Detailed XPS analysis

**Fig. 4:**
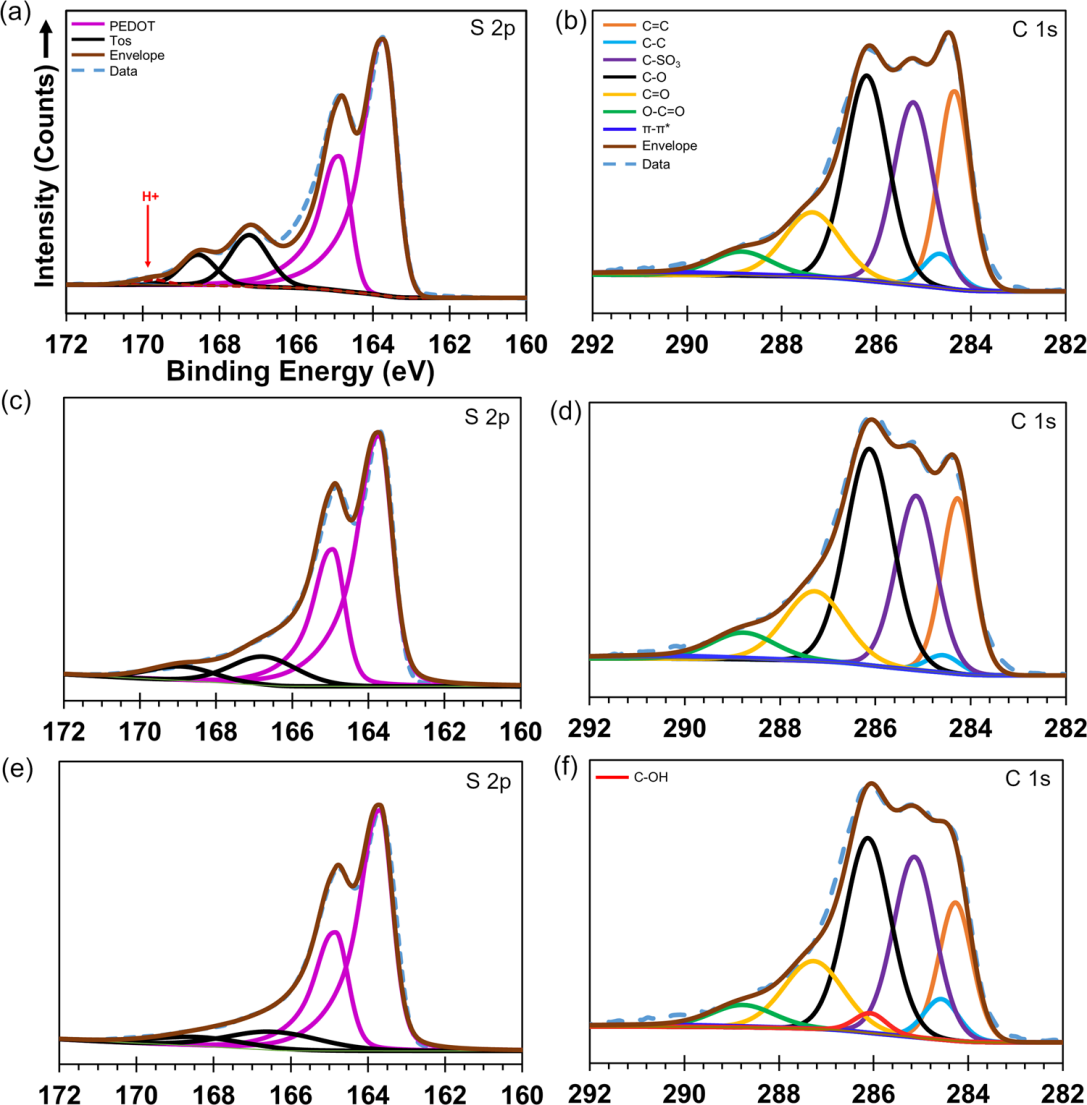
X-ray Spectroscopic analysis of doping in ER PEDOT:Tos. S 2p and C 1 s of **a**, **b** ER PEDOT:Tosylate, **c**, **d** PEDOT:Tos-NO_3_, and **e**, **f** PEDOT:Tos-OH.

To understand this in more detail, S 2p and C 1 s XPS fine scans were analysed for ER PEDOT:Tos, PEDOT:Tos-NO_3_ and PEDOT:Tos-OH (Fig. 4). The S 2p analysis shows that the Tos^−^ concentration (167.0 and 168.5 ev) relative to S in PEDOT (163.3 and 164.5 eV), the Tos^−^ doping level, decreases upon exposure to both NO_3_^−^ and OH^−^. For NO_3_^−^ exposure, even though the polymer is oxidised (and electrical conductivity enhanced) there is some ion exchange between the NO_3_^−^ and Tos^−^. In the case of OH^−^ ion exchange may also take place (as suggested by Khan et al.^26^), while also the amount of residual Tos^−^ being less than in PEDOT:Tos-NO_3_.

**Fig. 5:**
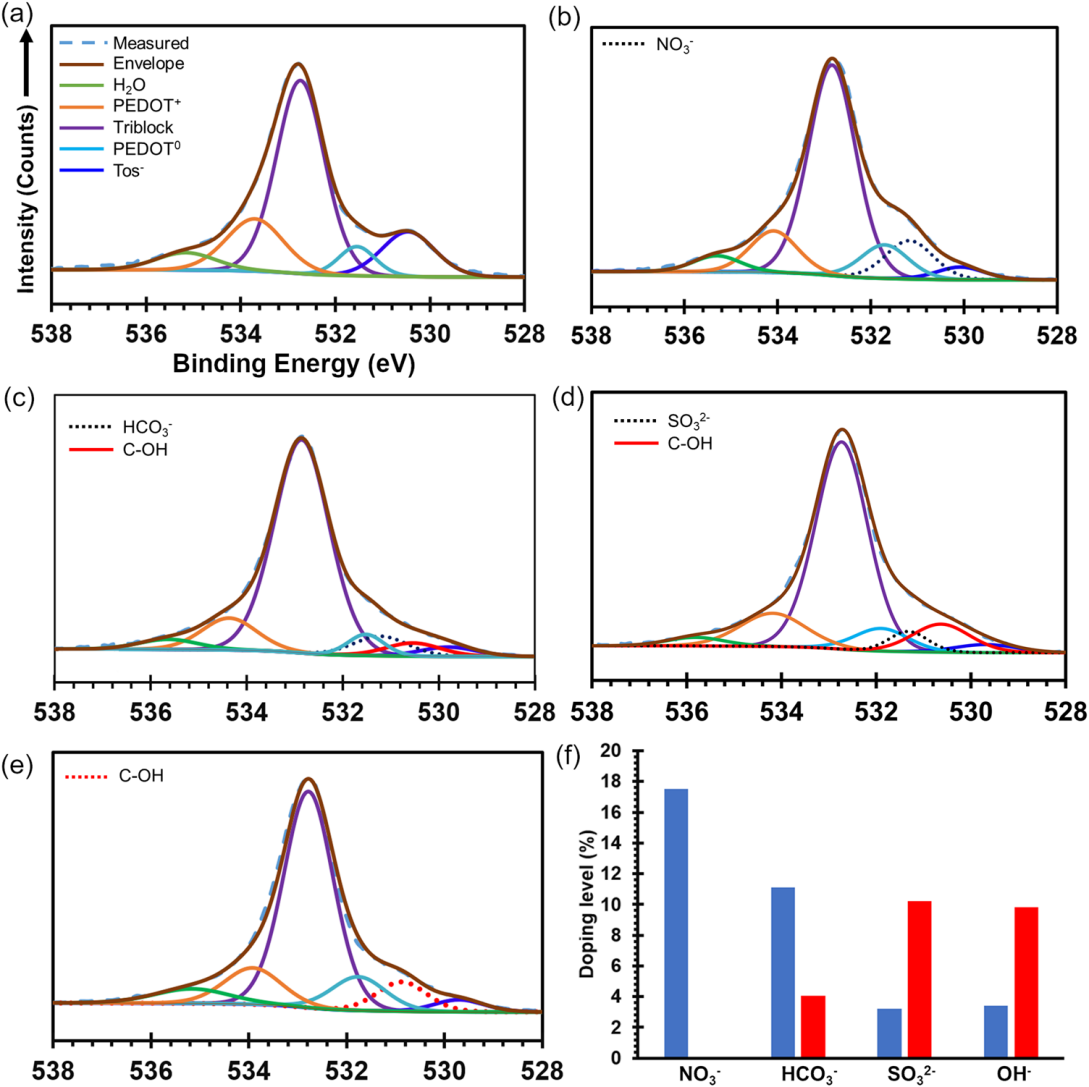
X-ray spectroscopic analysis of O 1 s to determine presence of OH. XPS analysis of O 1 s spectra of **a** ER PEDOT:Tos, and PEDOT:Tos-X after exposing to 1 mM **b** NO_3_^−^, **c** HCO_3_^−^ **d** SO_3_^2−^ and **e** OH^−^. The component fit for PEDOT^+^ and PEDOT^0^ refer to charged and neutral PEDOT, respectively. **f** The concentration of Tos^−^ (blue) and OH^−^ (red) ions in PEDOT:Tos-X for different anions defined from their S 2p and O 1 s fine scans respectively.

The fine scan of C 1 s spectra contains signatures of several different components within the final polymer layer; PEDOT, Tos^−^, and the triblock copolymer additive. Upon exposure of the ER PEDOT:Tos to NO_3_^−^, there is a decrease in the intensity of the peak related to aliphatic carbon (284.5 eV) within PEDOT, indicative of a more oxidised polymer. To achieve good agreement of the deconvoluted peaks with the experimental data, the C 1 s of PEDOT:Tos-OH requires addition of the peak at 285.9 eV related to C–OH bonds (Fig. 4f), though the presence of this bond becomes more evident in O 1 s spectra presented later (Fig. 5). The poor fit to the measured fine scan for PEDOT:Tos-OH when the 285.9 eV component is absent is provided in Supplementary Fig. 3. This chemical bond is a signature of possible hydrolysis of the carbocation of PEDOT; either via direct bonding of OH^−^ or stepwise via an oxonium ion intermediate. Prior work by Fabretto and co-workers observed a very similar bonding of amine-based compounds to VPP PEDOT:Tos^40^, where the neutral amine molecules acted as nucleophiles to bond to PEDOT carbocations. Recently van der Pol et al. studied the interaction of PEDOT:PSS with a series of aliphatic polyamines to enhance the resultant de-doping of the polymer by reacting with nucleophiles^41^. Bubnova et al. utilised the mechanism of de-doping with tertiary polyamines to enhance the thermoelectric properties of PEDOT:Tos^14^. This mechanism draws parallels with the earlier studies using tertiary amines (1,4-Diazabicyclo[2,2,2]octane) to react with carbocations (i.e. Crystal Violet, Malachite Green, tri-p-anisylmethyl, etc.) to understand the nature of the base-catalysis hydrolysis of carbocations^24,42,43^. To the best of our knowledge there has been no reports of conducting polymers interacting with water in the same manner as the amines. To further substantiate the presence of C–OH at 285.9 eV, the analysis of the O 1 s fine scan was undertaken.

The analysis of the O 1 s spectra of ER PEDOT:Tos, PEDOT:Tos-HCO_3_, PEDOT:Tos-SO_3_, PEDOT:Tos-CO_3_ and PEDOT:Tos-OH displayed the peaks corresponding to oxidised PEDOT (PEDOT^+^ at 533.70 eV) and neutral PEDOT (PEDOT^0^ at 531.50 eV), Tos^−^ (530.66 eV), the PEG-PPG-PEG triblock copolymer (532.8 eV) and water (535.60 eV). The presence of peaks related to water (still present despite the ultrahigh vacuum in the XPS, *ca*. 10^−8^ mbar) in all samples may arise from hydration of the PEG-PPG-PEG triblock copolymers within the polymer matrix. Interestingly, peaks related to OH groups (530.87 eV)^44^ were observed in varying concentrations across four of the samples. In the specific case of PEDOT:Tos-HCO_3_ the polymer film has an elevated electrical conductivity relative to ER PEDOT:Tos, commensurate with oxidation, yet shows the presence of hydroxylation in the XPS analysis. This indicates that hydrolysis of the carbocation occurs more broadly than first postulated based upon electrical conductivity measurements. Hydrolysis is occurring to an observable level where the pKa of the anion’s conjugate base is >6.4. In the specific case of PEDOT:Tos-HCO_3_ the doping (creation/stabilisation of carbocations) with HCO_3_^−^ and Tos^−^ outweighs neutralisation of carbocations from the hydrolysis.

### Reversibility of hydrolysis and oxidation

**Fig. 6:**
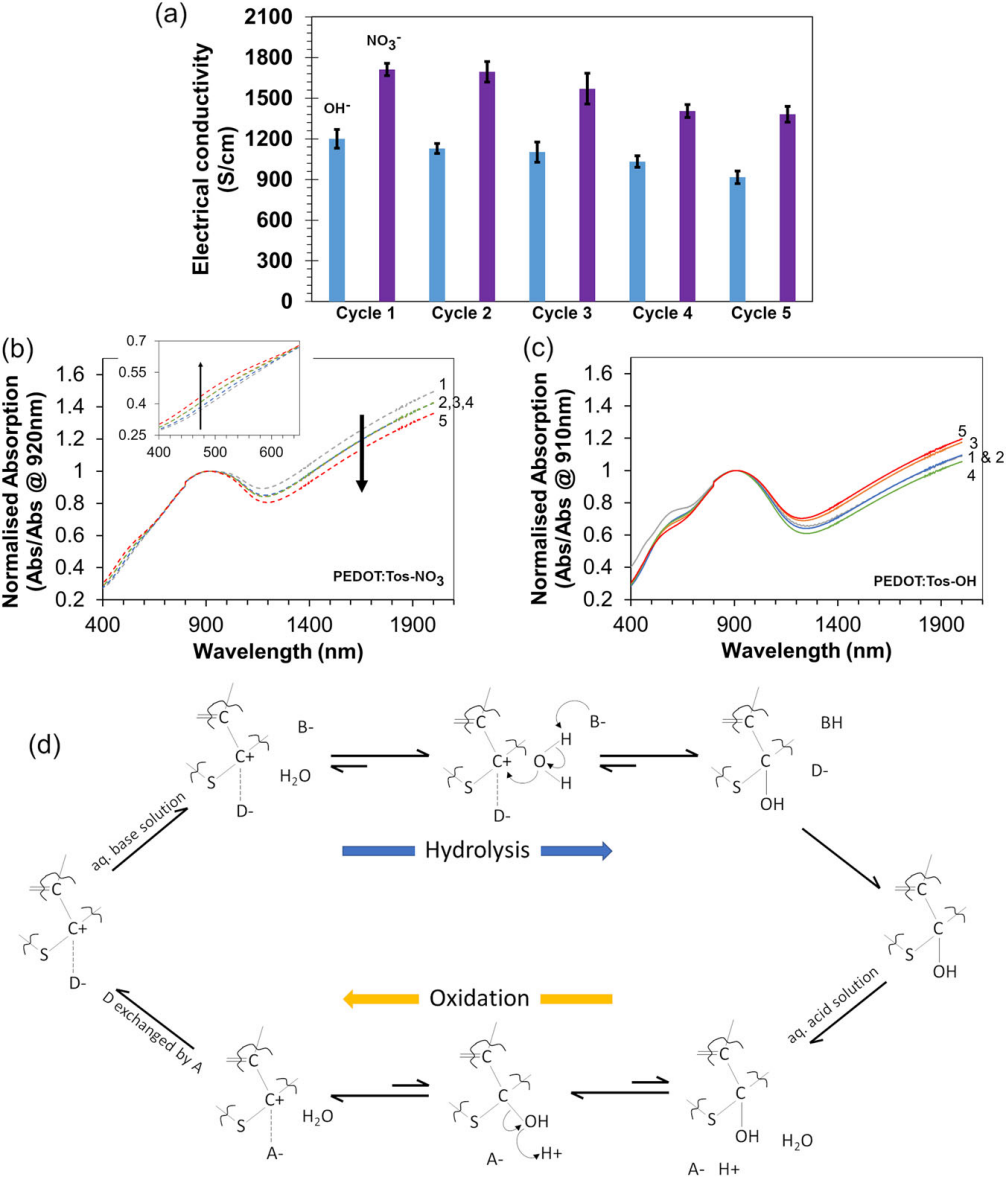
Reversibility and cyclability of hydrolysis and oxidation of PEDOT:Tos. **a** Electrical conductivity of PEDOT:Tos-X repeatedly cycled between 1 mM OH^−^ and 1 mM NO_3_^−^. Error bars represent ± one standard deviation of the electrical conductivity across multiple samples. This corresponds to a (normalised) change in optical absorption in the Vis-NIR spectra between **b** PEDOT:Tos-NO_3_ and **c** PEDOT:Tos-OH. The inset in **b** highlights the change in absorption in the Vis region related to neutral PEDOT. **d** The change in electrical and optical properties is rationalised when PEDOT doped with the primary dopant (D^−^) undergoes base (B^−^) catalysed hydrolysis yielding PEDOT-OH, and then acid (A^−^) catalysed dehydrolysis yielding oxidised PEDOT:A.

One aspect of the hydrolysis of carbocations is a reversible reaction converting alcohols to the carbocation (carbonium ion)^45,46^. To examine the reversible nature of the hydrolysis in these conducting polymer systems, ER PEDOT:Tos was cycled between solutions of NaOH and NaNO_3_. Each exposure leads to a change in the optical and electrical properties of the PEDOT:Tos-X (see Fig. 6). Between each exposure cycle there is a decrease or increase in electrical conductivity (Fig. 5a) for exposure to OH^−^ and NO_3_^−^, respectively. In parallel, the normalised optical absorption for PEDOT:Tos-X increases in the IR region and decreases in the 400–600 nm region for NO_3_^−^ exposure (Fig. 6b), and vice versa for OH^−^ exposure (Fig. 6c). The mechanism of hydrolysis to form PEDOT:Tos-OH and then re-oxidation to form PEDOT:Tos-NO_3_ is presented in Fig. 6d.

At rough approximation the hydrolysis-oxidation can be considered reversible, though observed reduction in the magnitude of the conductivity measured after each successive cycle suggests presence of non-reversible site reactions leading to degradation of the polymer. For PEDOT:Tos-NO_3_ the consequent reduction in electrical conductivity comes with the decreasing normalised absorption in the IR region (arrow in Fig. 6b), and increase in absorption in the 400–600 nm region (Fig. 6b inset). Conversely, there is no apparent trend in the normalised optical absorption of PEDOT:Tos-OH upon cycling (Fig. 6c). The DFT studies performed by Zozoulenko and co-workers to understand the optical properties of PEDOT highlights the complexity of interpreting optical spectra with regards to the polarons and bipolarons within^25^. That is, absorption peaks for polarons and bipolarons may occur at a range of different wavelengths—not necessarily following the convention that the peak in the 700–950 nm region is solely polarons, and absorption >1200 nm is bipolarons. What can be understood from the spectra in Fig. 6b is that each time PEDOT:Tos-NO_3_ is formed, there is a decrease in the number of charge carriers (lower IR absorption) and an increase in neutral chains (increased absorption in 400–600 nm region). For PEDOT:Tos-OH (Fig. 6c) the ratio of neutral to charged species varies in an almost random manner. Comparing this with the PEDOT:Tos-NO_3_ phase of the cycling highlights that there are a relatively lower number of charge carriers upon exposure to OH (again, relatively higher absorption at shorter wavelengths and lower IR absorption).

## Conclusion

In summary, hydrolysis of the prototypical conducting polymer PEDOT:Tos has been observed. Exposure of electrochemically reduced PEDOT:Tos to a range of select anions in aqueous solution results in the observation of hydroxylated PEDOT – dependent on the pKa of the anion’s conjugate acid. With high conjugate pKa values the hydroxylated PEDOT corresponds to relatively lower electrical conductivity and greater visible absorption; similar to what is obtained for electrochemically reduced PEDOT. At the other extreme (low conjugate pKa), the PEDOT:Tos is oxidised due to solution anions doping the polymer. A transition between oxidation and hydrolysis occurs near the pKa of 6.4. The experimental results and interpretation highlight that the base properties of the anions play a dominant role in the interaction with conducting polymers; more so than their geometry, size, charge or solubility. By using mixtures of anions (at constant solution pH and molar concentration) the hypothesis of general base-catalysed hydrolysis of conducting polymers is generated. Understanding and controlling the hydrolysis of doped conducting polymers is therefore critical for applications that exposes these materials to waters with at least moderate ionic strengths (biology, environment, etc).

## Methods

EDOT monomer and triblock copolymer (PEG-PPG-PEG; trade name P123) with a molecular weight of ~5800 Da was purchased from Sigma Aldrich and used as received. The water-soluble salts, sodium tosylate (NaTos), sodium nitrate (NaNO_3_), sodium chlorate (NaClO_3_), sodium sulfite (Na_2_SO_3_), sodium hydroxide (NaOH), sodium bicarbonate (NaHCO_3_), sodium carbonate (Na_2_CO_3_) and sodium phosphate (Na_3_PO_4_) were used as received from Sigma-Aldrich. The oxidant solution, Fe(III) tosylate (FeTos_3_) of 54 wt.% in n-butanol (trade name CB-54) was used as received from Heraeus, Germany.

The oxidant solution was prepared by adding the triblock copolymer to the CB-54 solution (FeTos_3_ in butanol) and diluting with ethanol. This resulted in an oxidant solution containing 257 mM FeTos_3_ and 58 mM triblock copolymer in a 2.6:1 vol/vol mixture of ethanol to butanol as the solvent carrier. This oxidant solution was spin cast onto air plasma treated (Diener Nano, 200 W RF air plasma at 0.4 mbar, 2 min treatment) glass substrates of 75 × 50 mm. The oxidant coated substrates were converted to PEDOT:Tos thin films using the VPP technique under vacuum condition at 45 mbar for 60 mins (using a VD115L vacuum oven from Binder, Germany), with the EDOT heated to 35 °C and the chamber at ambient temperature (22 °C)^36^. After polymerisation, VPP PEDOT:Tos thin films were rinsed in an ethanol bath to remove unreacted and/or residual oxidant material.

### Electrochemical reduction of PEDOT:Tos

The samples of VPP PEDOT:Tos were reduced electrochemically using the three-electrode setup (Voltalab PGZ100). The VPP PEDOT:Tos on the glass slide acted as the working electrode (WE), and Ag and Pt wires as the reference and auxiliary electrode. An aqueous solution of 500 mM NaTos was used as the electrolyte to electrochemically remove Tos from the PEDOT at an electrochemical potential of −1.0 Vdc for 15 s. These samples are referred to as ER PEDOT:Tos.

### Chemical exposure of ER PEDOT:Tos

The ER PEDOT:Tos samples were then exposed to different 1 mM aqueous sodium-based salt solutions for 24 h at 22 °C as part of the experimental studies. After exposure, the samples are referred to as PEDOT:Tos-X, where X represents the anion present in the salt solution.

In one set of experiments, ER PEDOT:Tos was exposed to 1 mM NaOH, characterised, and then exposed to 1 mM NaNO_3_ and re-characterised. Subsequently, this process was repeated 5 times to demonstrate the cycling of the PEDOT between PEDOT:Tos-OH and PEDOT:Tos-NO_3_. Each step followed the above procedure of 24 h exposure at 22 °C. After each exposure step, the samples were rinsed in Milli-Q water, by dipping into a vial of Milli-Q water and immediately removing, to remove any precipitated salt from the surface of the samples.

### Thickness measurements

Thickness (t) of the samples were measured by a Bruker Dektak XT profilometer. For measuring the thickness on the VPP PEDOT thin films, profiles were measured across a scratch in the polymer layer (scan length of 1000 μm and scan duration of 20 s, using the piezo setting for a 6.5 μm height). The scan was performed using a stylus force equivalent to the mass of 3 mg and the stylus of 12.5 μm in radius. A lateral scan resolution of 0.66 μm/point was used for all the measurements.

### Electrical conductivity measurements

A four-point probe from Jandel instruments, U.K. were used to measure the sheet resistance of the PEDOT, where these films are having uniform thickness. A van der Pauw measurement was carried out to calculate the sheet resistance (*R*_s_) of the samples by applying a constant current of 3 mA and recording the potential between the electrodes. By using (σ = 1/*R*_s_ × t), the electrical conductivity (σ) of the films were calculated.

### X-ray photoelectron spectroscopy

All ER PEDOT:Tos and PEDOT:Tos-X samples were rinsed in Milli-Q water, by dipping into a vial of Milli-Q water and immediately removing, to remove any precipitated salt from the surface of the samples. This rinsing process was minimised to avoid unwanted reactions with water, but to still ensure removal of precipitated salt that could contaminate the XPS measurements. All spectra were acquired using a Kratos Axis Ultra DLD spectrometer. The x-ray source used was an Al monochromatic Kα source with energy 1486.6 eV. Power output of the source was 225 W obtained by using source parameters 15 kV and 15 mA. The analysis chamber vacuum during spectral acquisition was 4 × 10^−8^ mbar. The charge neutraliser (Kratos charge neutraliser) was turned on and operating with neutraliser parameters: filament current 2.0 A, charge balance 3.6 V and filament bias 3.5 V. Instrument calibration used metallic gold and copper. The work function was set to −1.441 V giving an Au 4f_7/2_ binding energy position of 84.03 eV. The spectrometer dispersion was set so that Cu 2p_3/2_ was located at the binding energy of 932.69 eV. Instrument calibration procedure was according to AS ISO 1547:2006-Surface chemical analysis-X-ray photoelectron spectrometers-calibration of spectrometer energy scale. The resulting metallic Ag 3d_5/2_ was found to be located at 368.26 eV. All spectra were collected over a rectangular analysis area with dimensions 300 × 700 µm. Wide scan spectra were collected using the pass energy 160 eV. Narrow scan spectra were collected with a pass energy of 20 eV. Spectra were charge corrected to adventitious carbon located at 284.8 eV. All spectra were analysed using CasaXPS version 2.3.18PR1.0 (Casa Software Ltd, Wilmslow, Cheshire, UK).

The spectral shape of the C 1s, S 2p and O 1s were aligned with C-C (hydrocarbon) peak at 284.8 eV. In the S 2p spectra, two doublets were used for fitting with the hybridization of the 2p orbital as 1.21 (±0.5) eV and the area of S 2p_1/2_ being half that of S 2p_3/2_, respectively^47^. The asymmetric line shape is used for fitting the S 2p spectra of PEDOT chain defining the metallic behaviour of the delocalised positive charges across the polymer chains as was suggested by Zotti et al.^48^.

The specific fitting parameters for S 2p (Table 1), O 1s (Table 2) and C 1s (Table 3) are as follows;

**Table 1:** XPS fitting parameters for S2p.

Components	Line shape	FWHM	Binding energy (eV)
PEDOT (S 2p_1/2_)	SGL (30) T(1)	0.82	168.70
PEDOT (S 2p_3/2_)	SGL (30) T(1)	0.82	164.50
Tos (S 2p_1/2_)	GL (30)	1.2	166.92
Tos (S2p_1/2_)	GL (30)	1.2	168.12

**Table 2:** XPS fitting parameters for O1s.

Components	Line shape	FWHM	Binding energy (eV)
Tos^**−**^	GL (30)	1.5	530.66
PEDOT^**0**^	GL (30)	1.25	531.50
Triblock	GL (30)	1.25	532.75
PEDOT^+^	GL (30)	1.5	533.70
H_2_O	GL (30)	1.5	535.60
NO_3_^−^	GL (30)	1.2	531.17
HCO_3_^−^	GL (30)	1.2	531.21
SO_3_^2−^	GL (30)	1.2	530.63
OH^−^	GL (30)	1.2	530.87^49^

**Table 3:** XPS fitting parameters for C1s.

Components	Line shape	FWHM	Binding energy (eV)
C=C	GL (30)	0.87	284.26
C–C	GL (30)	0.87	284.56
C–SO_3_	GL (30)	1.0	285.13
C–OH	GL (30)	0.87	286.03
C–O	GL (30)	1.0	286.11
C=O	GL (30)	1.4	287.27
O–C=O	GL (30)	1.5	288.76
pi–pi*	GL (30)	3.0	291.10

### X-ray diffraction

X-ray diffraction measurements were made for ER PEDOT:Tos, PEDOT: Tos-NO_3_, PEDOT:Tos-ClO_3_, PEDOT:Tos-SO_3_, PEDOT:Tos-CO_3_, and PEDOT:Tos-OH. All ER PEDOT:Tos and PEDOT:Tos-X samples were rinsed in Milli-Q water, by dipping into a vial of Milli-Q water and immediately removing, to remove any precipitated salt from the surface of the samples. These measurements were conducted on a Malvern Panalytical (Empyrean) X-ray diffractometer with Cu Kα radiation (*λ* = 0.154 nm). The receiving slit size and step size of 0.1 mm and 0.007 (2ϴ) were used for the analysis; all the samples were obtained during analysis at 23 °C.

## Supplementary information

Supplementary Information

## Data Availability

The authors declare that the data supporting the findings of this study are available within the paper and its Supplementary Information files.
