# Supplementary material for: Hydrolysis of doped conducting polymers
Source: Commun Chem. 2020 Nov 4;3:153. doi: 10.1038/s42004-020-00404-y (PMC9814606; doi:10.1038/s42004-020-00404-y)
Supplement: Supplementary file 1 — Supplementary Information [file 42004_2020_404_MOESM1_ESM.pdf]

Hydrolysis of doped conducting polymers

Vithyasaahar Sethumadhavan[1], Kamil Zuber[1], Peter Teasdale[2], Christopher Bassell[1], Drew Evans[1]*

[1]Future Industries Institute, University of South Australia, Mawson Lakes, South Australia, 5095 Australia

[2]UniSA STEM, University of South Australia, Mawson Lakes, South Australia, 5095 Australia

*Corresponding author: email – Drew.evans@unisa.edu.au

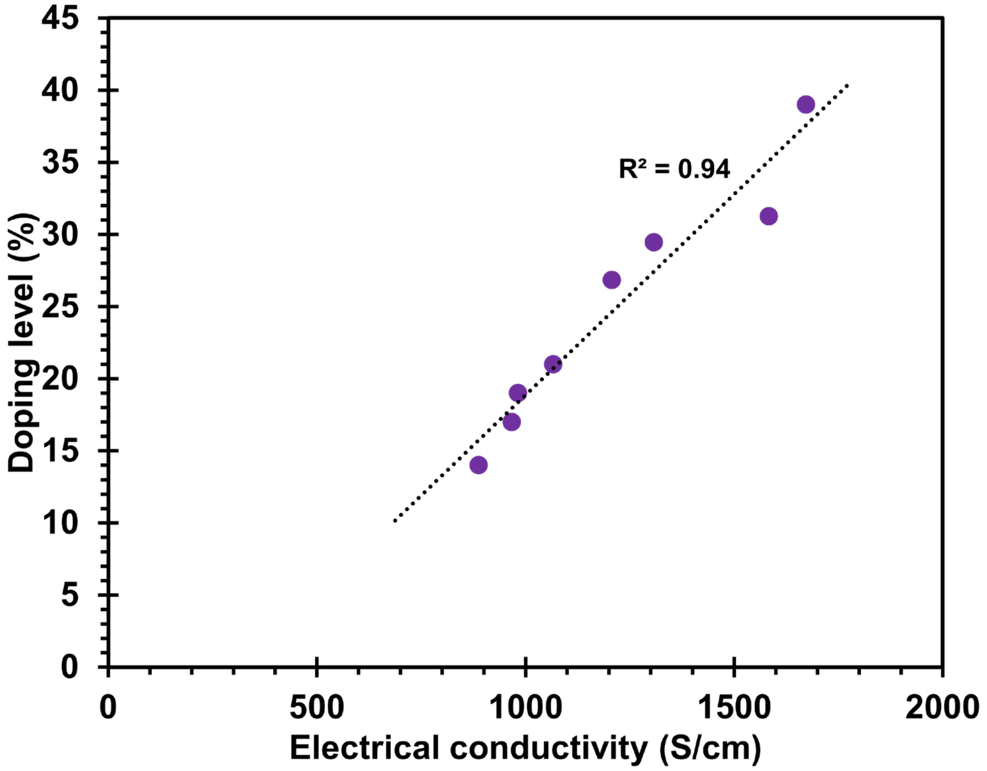

Supplementary Figure 1. Linear correlation of Doping level and Electrical conductivity for PEDOT:Tos-X samples. The doping level determined via XPS is compared to the electrical conductivity for each sample.

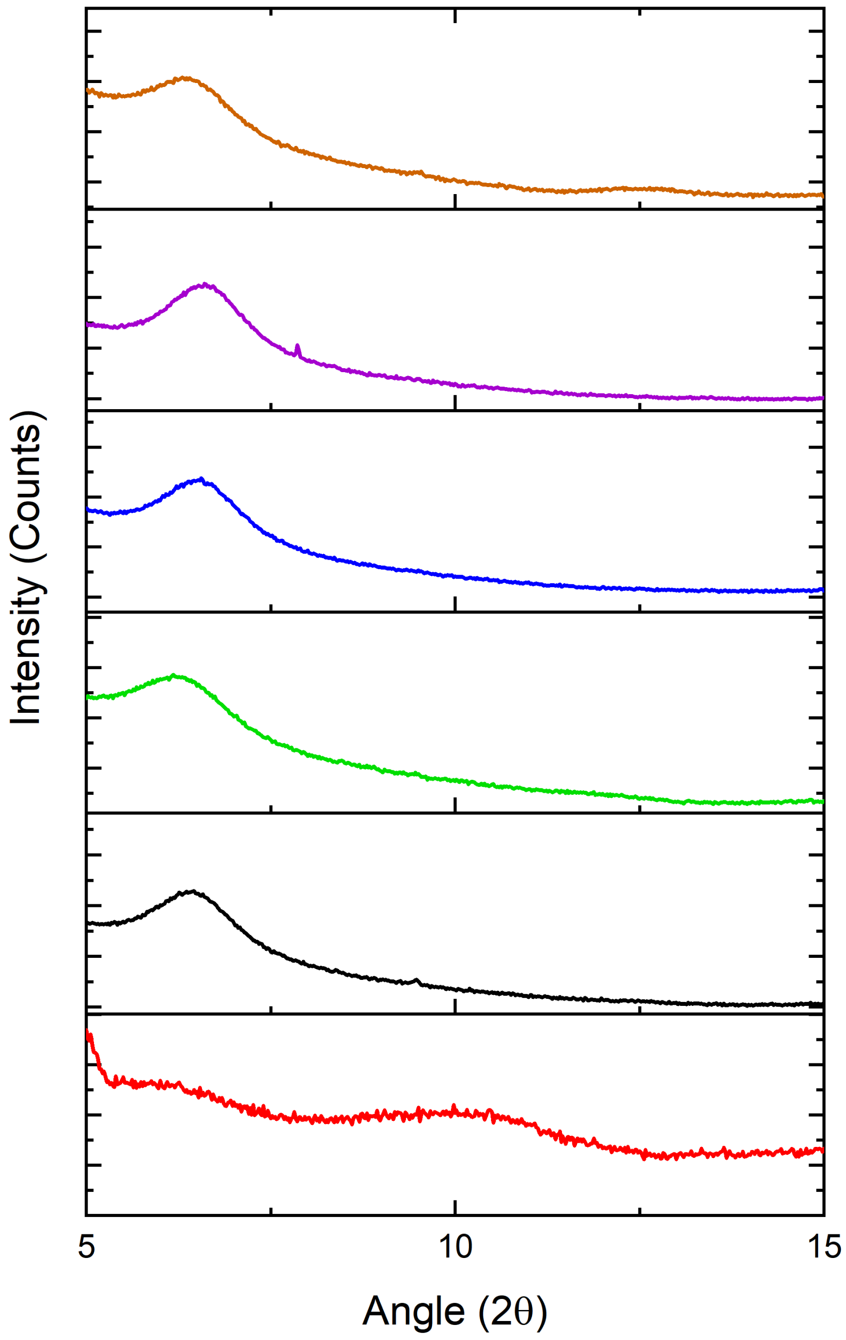

Supplementary Figure 2. X-ray diffractograms for PEDOT:Tos-X samples. Samples of ER PEDOT:Tos (brown) and PEDOT:Tos-X [NO_3^- = purple; ClO_3^- = blue; SO_3^{2-} = green; CO_3^{2-} = black; OH^- = red]. The primary diffraction peak for the d(100) reflection (at approximately 7 degrees) is used to calculate the crystallite domain size.

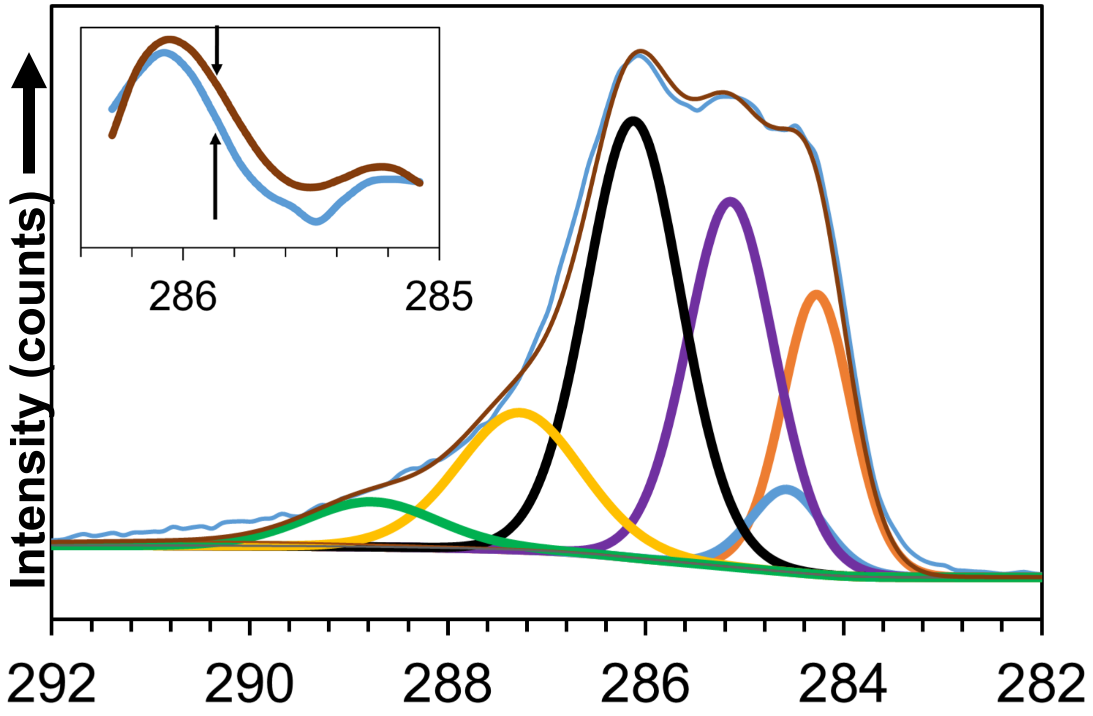

Supplementary Figure 3. XPS C 1s fine scan for PEDOT:Tos-OH without the C-OH component. *The absence of this component results in poorer agreement of the fitting envelope to the measured data (highlighted in the inset).*
